# Recurrent Implantation Failure-update overview on etiology, diagnosis, treatment and future directions

**DOI:** 10.1186/s12958-018-0414-2

**Published:** 2018-12-05

**Authors:** Asher Bashiri, Katherine Ida Halper, Raoul Orvieto

**Affiliations:** 1 Recurrent Pregnancy Loss Clinic, Maternal-Fetal Medicine, and Ultrasound, Soroka University Medical Center, P.O.B. 151, 84101 Beer Sheva, Israel; 2 Faculty of Health Sciences, Ben-Gurion University of the Negev, P.O.B. 151, 84101 Beer Sheva, Israel; 3 Department of Obstetrics and Gynecology, Chaim Sheba Medical Center, Tel-Hashomer, 52621 Ramat Gan, Israel; 4 Sackler Faculty of Medicine, Tel-Aviv University, Tel Aviv, Israel

**Keywords:** Recurrent implantation failure-RIF, Low molecular weight heparin, Endometrial scratching, Chronic endometritis, Preimplantation Genetic Screening- PGS, IVIG, Progesterone, Reward system

## Abstract

Recurrent implantation failure (RIF) refers to cases in which women have had three failed in vitro fertilization (IVF) attempts with good quality embryos. The definition should also take advanced maternal age and embryo stage into consideration. The failure of embryo implantation can be a consequence of uterine, male, or embryo factors, or the specific type of IVF protocol. These cases should be investigated to determine the most likely etiologies of the condition, as this is a complex problem with several variables. There are multiple risk factors for recurrent implantation failure including advanced maternal age, smoking status of both parents, elevated body mass index, and stress levels. Immunological factors such as cytokine levels and presence of specific autoantibodies should be examined, as well as any infectious organisms in the uterus leading to chronic endometritis. Uterine pathologies such as polyps and myomas as well as congenital anatomical anomalies should be ruled out. Sperm analysis, pre-implantation genetic screening and endometrial receptivity should be considered and evaluated, and IVF protocols should be tailored to specific patients or patient populations. Treatment approaches should be directed toward individual patient cases. In addition, we suggest considering a new initial step in approach to patients with RIF, individualized planned activities to activate the brain's reward system in attempt to improve immunological balance in the body.

## Background

The breakthrough of assisted reproductive technology (ART) in recent scientific history has allowed couples previously unable to conceive to achieve viable pregnancy, while at the same time opening a new window into detection of early miscarriages. Although ART has improved outcomes for struggling couples, a new challenge has emerged: recurrent implantation failure (RIF). Multiple failed cycles can leave couples frustrated and desperate for explanations. It is necessary to determine the etiologies of RIF in order to propose new and beneficial solutions for these patients. These processes are distinctly different from recurrent pregnancy loss (RPL) which is a disorder defined by the ASRM, as the loss of two or more consecutive clinical pregnancies until 20 weeks gestation [1] and by the ESHRE as two or more pregnancy losses until 24 weeks gestation (including chemical pregnancy) [2]. Around 5% of women are expected to suffer from two consecutive pregnancy losses, almost 75% are due to an implantation failure, and therefore are never recognized as clinical pregnancies [3].

This review aims to examine biochemical pregnancy, RIF, and the related issues in patients undergoing ART. Our goal is to examine the causes, potential treatments, and recommendations for patients suffering from these conditions. Since many different etiological factors may lead to RIF, our goal is to establish a standardized assessment and course of action for these patients prior to applying personalized interventional approaches. In addition, we aim to evaluate biochemical pregnancy rates, specifically in recurrent implantation failure patients undergoing ART, and to determine whether this patient group is unique with different requirements for assessment and treatment.

### Definitions: RIF and Biochemical pregnancy

The term ‘implantation failure’ can be used to describe both patients who have never shown quantifiable signs of implantation such as increased levels of hCG, and those who have increased hCG production without later ultrasound evidence of a gestational sac [4]. Implantation failure can apply to patients undergoing assisted reproductive technology (ART) and patients trying to conceive without any fertility treatment.

Recurrent implantation failure (RIF) is only applicable to patients undergoing ART. Although there is no accepted formal definition for recurrent implantation failure, Orvieto suggests that it is after three failed in vitro fertilization-embryo transfer (IVF-ET) cycles with good quality embryos transferred [5]. Zeyneloglu et al. agree that it is after three unsuccessful cycles of IVF specifically with two embryos of high quality [6]. Simon and Laufer add that the embryo and the endometrium can both play an active role in RIF [7]. It is also important to consider maternal age in the definition, and whether the embryos were transferred at the cleavage-stage or as blastocysts [8]. Coughlan et al. suggest a more complete working definition taking into account maternal age, number of embryos transferred, and number of cycles completed. They define RIF as the failure of clinical pregnancy after 4 good quality embryo transfers, with at least three fresh or frozen IVF cycles, and in women under the age of 40 [4].

Biochemical pregnancy is defined in similar terms by a variety of authors. Maesawa uses the International Committee Monitoring Assisted Reproductive Technologies and World Health Organization definition for biochemical pregnancy as the detection of hCG in blood or urine without subsequent clinical signs of pregnancy [9]. Coulam defines biochemical pregnancy when two or more increased values of hCG can be measured, yet there can be no evidence of gestational sac detected on transvaginal ultrasound 2 weeks later. Therefore, only a chemical measure indicates that a pregnancy occurred. By previous definition, biochemical pregnancy falls into the category of implantation failure [10]. This differs from a clinical pregnancy which is determined, as defined by the American Society of Reproductive Medicine (ASRM), by ultrasound examination with evidence of a gestational sac or by histopathological examination [1].

Despite the fact that many authors agree on a similar general definition, the parameters used to measure the hCG level differ substantially between > 5 to > 25 mIU/ml [11–13].

### Incidence

Due to variations in definitions for recurrent implantation failure and biochemical pregnancy, there is scarce data that accurately represents the incidence or prevalence. Biochemical pregnancy is actually not uncommon, and its reported incidence varies from 8 to 33% in the general population, including those who spontaneously conceived [9]. However, it is not clear how accurate these figures are since most patients who recognized that they had a biochemical pregnancy were undergoing ART. Therefore, it is likely that these patients are measuring their hCG levels earlier than those who are conceiving spontaneously and waiting until they a miss a period before taking a pregnancy test [14]. In spontaneous conception it is estimated that 30% of pregnancies are lost before implantation and 10% are clinical pregnancy losses [15]. It is also important to note that spontaneous pregnancy is only achieved in around 30% of normal fertile couples on the first try, and many succeed on subsequent efforts [16]. Moreover, it may be worth considering whether or not biochemical pregnancy is a pathological process.

## Risk factors

### Maternal age

Maternal age plays a crucial role in the quality of the embryos that are used for IVF [4]. It has long been known that as maternal age increases so does the frequency of aneuploidy [6]. As a result, many authors have age cut offs in their studies. Pregnancy rates also have been found to be decreased as maternal age increases [11]. In particular, Salumets et al. found that the major predictive factor contributing to pregnancy outcome in frozen embryo transfer, specifically with Intracytoplasmic Sperm Injection (ICSI) technique, was maternal age. The patient’s age at oocyte collection and freezing was the sole determining factor for biochemical pregnancy outcome. Starting around age 39, there was a significantly higher rate of occurrence of biochemical pregnancy. Delivery rate is also affected by both maternal age and embryo quality [17]. Shapiro et al. found that there are higher rates of embryo-endometrial asynchrony with increasing maternal age. 50% of transfers were asynchronous in women < 35 years old in comparison with 68.1% which were asynchronous in women > 35 years old. In addition, implantation rates, which were calculated for each blastocyst transfer and therefore treated as a numeric value, were significantly lower in IVF cycles in women of age ≥ 35 (mean 24.5 ± 36.8) compared with women < 35 (mean 41.1 ± 42.1), and biochemical pregnancy rates were significantly higher in women ≥35 compared to < 35 (28.1% vs. 14.9% respectively). Live birth rate was significantly higher in women < 35 (50.7%) than in women > 35 (28.5%) Patients > 35 years old also had reduced oocyte yield, blastocyst formation, and endometrial thickness [18]. The United States Center for Disease Control and Prevention reports overall ART success rates each year, with the most recent statistics currently from 2015. In agreement with the literature, implantation rates in both fresh and frozen embryo transfer in patients < 35 (41.3% and 47.1%) were dramatically higher than those rates in women > 44 (1.9% and 16.2%). It was interesting to note that the implantation rate when using donor oocytes, representing all age categories, was 53.6% in fresh embryo transfers and 40.2% in frozen embryo transfers [19]. This further supports the idea that embryo quality including genetic characteristics, declines with increasing maternal age.

### BMI

Increased BMI (> 25 kg/m^2^) has also been shown to impact implantation rate [20]. In patients undergoing IVF, Class I, II, and III obese patients (BMI > 30 kg/m^2^) had the highest chance of implantation failure demonstrated by respective odds ratios, 0.69 (0.53–0.90), 0.52 (0.36–0.74), and 0.58 (0.35–0.96), when compared with patients of normal weight (BMI 18.5–24.99 kg/m^2^). Though there have been no reported differences across different BMI groups for biochemical pregnancy specifically, the Class III Obesity Patients (BMI > 40 kg/m^2^) had the highest overall rates of miscarriage (including biochemical pregnancy) [21]. In addition, overweight and obese women (BMI > 25 kg/m^2^) undergoing IVF who had fewer oocytes collected had higher risks of implantation failure and miscarriage than women of healthy weight with the same number of collected oocytes [20]. When more oocytes are collected, there is a higher chance of increased quality of embryo for transfer, and with fewer oocytes comes a higher likelihood of negative pregnancy outcome due to fewer good quality embryos. Obese women required more gonadotropin stimulation cycles, yet they had statistically fewer oocytes for collection (average of 8 vs. 10 in non-obese women, *P* = .03). This suggests that the oocyte quality and follicular development might be affected by obesity [22]. Though recurrent pregnancy loss is a different entity than recurrent implantation failure, elevated BMI is considered to be the most important risk factor, after increasing maternal age, in miscarriage for patients suffering from recurrent pregnancy loss [15].

### Smoking

It can be particularly difficult to find valid information on smoking’s impact on fertility due to the bias of self-reported smoking during pregnancy. This is largely due to the fact that women might be concealing their smoking since there is a negative attitude toward smoking in pregnancy [23]. Smoking has been shown to lead to a significantly increased risk of miscarriage (time unspecified) for each pregnancy in comparison with non-smoking patients undergoing ART [24]. In women undergoing IVF, smoking patients were found to have lower estradiol levels during ovarian stimulation. Cigarette toxins might play a role in disrupting corpus luteum formation and implantation of the embryo [23]. Fuentes et al. also demonstrated that female smokers with higher serum cotinine levels (a nicotine metabolite) had significantly fewer ova retrieved during IVF cycles, though cotinine levels had no significant impact on rates of implantation in IVF cycles [25]. In addition, smoking patients had a decreased live birth rate suggesting that smoking has an overall negative impact on pregnancy outcome [24]. Maternal smoking was more commonly linked to spontaneous miscarriage with normal fetal karyotype than abnormal karyotype, suggesting that the toxic effects of carbon monoxide and nicotine might be the principle factors causing harm. Carbon monoxide can cause a depletion of oxygen to the fetus, and nicotine can lead to vasoconstriction and decreased nutrients to the fetus due to maternal appetite suppression [26]. Pregnancy rates have been shown to be lower overall among smokers when compared with non-smokers, yet there are minimal differences in conception rates between the two groups [23]. It is also necessary to consider the effect of smoking on male fertility. Kunzle et al. found that male smokers had a significantly decreased sperm count (229.4 ± 251.5 × 10^6^ cells vs. 278.1 ± 264.2 × 10^6^ cells, *P* = .0001), higher percentage of abnormal morphology (21.2 ± 14.6% normal forms vs. 23.7 ± 15.5% normal forms, *P* = .0007) decreased motility (105.6 ± 132.7 × 10^6^ cells vs. 126.6 ± 136.8 × 10^6^ cells, *P* = .0016) and increased pH level measured by citrate concentration (86.7 ± 57.3 vs. 111.7 ± 303.1, *P* = .0072) [27].

### Stress

It has been shown that elevated levels of cortisol, also known as “the stress hormone,” lead to a 2.7 times greater chance (95% CI = 1.2–6.2) of miscarriage within the first 3 weeks after conception in comparison with women with low cortisol levels. Cortisol production in the body rises in response to psychological, immunological, and other stressors, suggesting that it serves as a marker signaling the female body that it is not in its best state for reproduction [28]. This suggests that preventing or decreasing maternal stressors may have a positive outcome on pregnancy. In contrast to this study, Pasch et al. found that psychological stress such as clinical anxiety or depression does not have a significant affect on IVF outcome in women undergoing a first time fertility treatment. However, it is IVF failure that may lead to higher rates of both anxiety and depression in the immediate period after a negative IVF outcome. There were higher rates of post-IVF depression in women with IVF failure than in women who achieved successful pregnancy (44% vs. 30% *P* < .001). IVF failure was also associated with higher rates of post-IVF anxiety in comparison with women who were able to become pregnant successfully (60% vs. 50% *P* < .001) [29]. It is important to consider that this study addressed only those undergoing first time fertility treatment, and that it was not necessarily those who would go on to suffer from recurrent implantation failure.

## Pathophysiological mechanisms of recurrent implantation failure

### Immunological

#### Natural killer cells – Peripheral and uterine

Yamada et al. evaluated pre-conception peripheral Natural Killer (NK) cell level and activity in women with normal fetal karyotypes suffering from RPL and the effects of those NK cells on future spontaneous pregnancy rates [30]. Natural Killer cells were given their name because of their ability to kill leukemic cell lines [31]. It was determined that the levels of peripheral NK cells were relatively equal in women whose next pregnancy resulted in both biochemical pregnancy and miscarriage, suggesting here that these immune cells might not affect only implantation but may also involve an additional mechanism in pregnancy. Threshold values for high NK cell activity (> 46%) and level (> 16.4%) were established by the receiver operating characteristics curve (a computer program), as those that pose a greater risk of biochemical pregnancy and miscarriage. In addition, the NK cell level and activity in these two groups were significantly higher than those of the women who went on to achieve successful live births [30]. Sacks et al. found that women with RIF had significantly increased peripheral NK cells by concentration (0.23 × 10^9^/L ± 0.11 vs. 0.20 × 10^9^/L ± 0.13) and percentage (> 18%, threshold value) of lymphocytes compared with controls. However, it is important to note that the sensitivity of this test was only 11% suggesting that women with RIF might have multiple other factors that contribute to their difficulty in achieving pregnancy. Testing NK cell levels therefore cannot necessarily be used to distinguish women with RIF from the general population, but rather might be used in women with already established RIF, to determine whether the etiology of their implantation failure is related to their immunological profile [32].

Uterine Natural Killer (uNK) cells are derived from the NK cell line due to their CD56+ marker, however they do not have the same ability to destroy cancerous cell lines and other HLA class 1 negative molecules. Therefore, they may not actually have a deleterious effect on an implanted embryo [31]. Due to similar phenotype, CD117 + CD94 − CD3−, it is likely that peripheral NK cells in stage 3 of their development migrate to the endometrium and complete their maturation and development there to become uNK cells [33]. In fact, they are the dominant immune cell type in the mucosa of the uterus and have been suggested to play a role in trophoblast invasion and increased spiral artery blood flow. It can also be difficult to measure uNK cells as their level varies throughout the menstrual cycle due to fluctuating progesterone and other hormone levels. As a result, any change in hormonal levels in healthy fertile women might affect uNK cell levels without having any impact on pregnancy outcome [31]. In 2014 a study published by Santillan et al. that both peripheral NK and Uterine NK cells are elevated in patients with RIF. Blood levels of NK cells were 13.4 ± 1.2% (range 2.63–29.01) in RIF patients and 8.4 ± 0.7% (range, 5.72–13.28) in controls. Uterine NK cells were measured via endometrial biopsy and levels greater than > 250 CD56 cells per high power field 400× were found in 53% of idiopathic RIF patients and only in 5% of controls. This allowed visualization of the uterine NK cells with immunohistochemical staining. Though cut off values still require standardization, analysis of NK cells might eventually prove to be useful to women suffering from idiopathic RIF [34]. On the other hand, a recent meta-analysis by Seshadri et al. set out to determine the role of both peripheral and uterine NK cells in infertility and recurrent miscarriage and found some conflicting data regarding their role. There was no significant difference in peripheral (SMD -0.33; 95% CI -1.06; 0.40; *P* = 0.37) and uterine (SMD -1.82; 95% CI -4.80; 1.17; *P* = 0.23) NK cell levels expressed as percentages between fertile and infertile women, though there were significantly higher levels of peripheral NK cells in the infertile women in studies in which they were expressed as numbers (SMD 3.16; 95% CI 1.07; 5.24; *P* = 0.003). In addition NK cells levels did not seem to have an association with live birth rate in those undergoing IVF (RR 0.57; 95% CI 0.06; 5.22; *P* = 0.62). There were also significantly higher peripheral NK cell percentages (SMD 1.36; 95% CI 0.04; 2.69; *P* = 0.04) and numbers (SMD 0.81; 95% CI 0.47; 1.16; *P* < 0.00001), however, there was no significant different in uNK cells levels between women with recurrent miscarriage and controls (SMD 0.40; 95% CI -1.24; 2.04; *P* = 0.0.63). It is unclear why there are differing results when NK cells measurements are expressed as percentages versus numbers [35]. To date these studies are still investigative, and the role of NK cells in recurrent implantation failure and recurrent miscarriage is still controversial. NK cell level and activity is just one aspect of the immune system involved in women suffering from infertility, and we need more data in order to yield clinical value from this information.

#### Th1/Th2 ratio and TNF-α levels

There is a relative agreement that elevated levels of Th1 cells are associated with rejection of embryos, whereas elevated Th2 cell levels are associated with pregnancy. These T cells are measured by their cytokine production [36]. Cytokines produced by Th1 cells, such as TNF-α, suppress trophoblastic growth and promote inflammatory and thrombotic responses in maternal uterine blood vessels thus adversely affecting implantation. Cytokines produced by Th2 cells such as IL-4, IL-6 and IL-10 inhibit Th1 cell induced tissue factor by monocytes. Higher mean ratios of TNF-α/IL-4 (15.96 ± 2.30 and 12.81 ± 2.52) and TNF-α/IL-10 (60.05 ± 8.63 and 48.67 ± 10.08) in peripheral blood samples have been measured in women with multiple implantation failures both with no history and history of spontaneous miscarriage compared with mean ratios in controls (TNF-α/IL-4, 9.49 ± 0.79 and TNF-α/IL-10, 29.45 ± 2.60) [37]. However, it is interesting to note that no biochemical pregnancies resulted in this sample population indicating that biochemical pregnancy may have other pathophysiological mechanisms.

#### Auto-antibodies

Several autoimmune antibodies such as anti-nuclear antibodies, anti-cardiolipin antibodies (ACAs), and anti-phospholipid antibodies are involved in biochemical pregnancy loss. There has been an increase in biochemical pregnancy in patients with these auto-antibodies, as well as morphological changes in the embryos themselves specifically in those women with ACAs. It has been proposed that there is a direct interaction between ACAs and the embryos prior to their implantation [38]. Although the mechanism is still not well understood, there is also a strong association between anti-β_2_ glycoprotein 1 and anti-nuclear antibodies and implantation failure. β_2_ glycoprotein 1 is the cofactor for anticardiolipin. Stern et al. found that 30% of patients with implantation failure tested positive for this antibody or another antiphospholipid antibody (Anti-phosphatidylinositol (aPI), Anti-phosphatidylethanolamine (aPE), Anti-phosphatidylserine (aPS)) in comparison with 16% of fertile controls (*P* = .019) [39]. It is important to understand that this is a strong association, but there is no evidence to suggest that these antibodies directly cause the implantation failure.

#### Antiphospholipid syndrome

Antiphospholipid syndrome (APS) and its definition might be important to consider in relation to RIF patients. According to the APS clinical criteria the patient must have vascular thrombosis or pregnancy morbiditiy (fetal death after 10 weeks, premature birth before 34 weeks, or three or more consecutive miscarriages before 10 weeks of gestation). Laboratory criteria can include either Lupus anticoagulant (LA) measured in the plasma twice and 12 weeks apart, Anticardiolipin antibody in plasma >40GPL or MPL or > 99th percentile, measured twice and 12 weeks apart or Anti-*β*_2_ glycoprotein-I antibody in plasma >99th percentile measured twice and 12 weeks apart. In order to be diagnosed with Antiphospholipid Syndrome, one clinical and one laboratory criteria must be met [40]. Studies have shown that these antibodies are present in RIF patients, however, these specific clinical and laboratory criteria might not be met in RIF patients. A meta analysis conducted by Hornstein et al. demonstrated that data from seven studies revealed no statistically significant association between presence of anti-phospholipid antibodies and clinical pregnancy or live birth rates in future IVF cycles, though exact levels were not specified [41]. There is accumulating evidence of non-criteria clinical and laboratory manifestations of APS, of which one criterion is two or more unexplained failed IVF cycles. In addition, some studies have suggested that these women benefit from standard APS treatment [42]. Due to presence of specific antibodies implicated in both APS and RIF, it is important to consider if RIF should be added to the clinical criteria for antiphospholipid syndrome.

#### Hereditary thrombophilia

There is some data suggesting that hereditary thrombophilias may be involved in a subgroup of women with unexplained recurrent implantation failure. Azem et al. found that there were higher rates of inherited thrombophilias such as methylene tetrahydrofolate reductase (MTHFR) deficiency, factor V leiden, prothrombin deficiency, and antithrombin III deficiency in women with RIF in comparison with controls (44% vs. 18.2%, *P* = .012) [43]. Though this needs to be confirmed by further studies, this could lead to the evaluation of antithrombotic agents as another potential intervention for women suffering from RIF.

### Infection

**Fig. 1:**
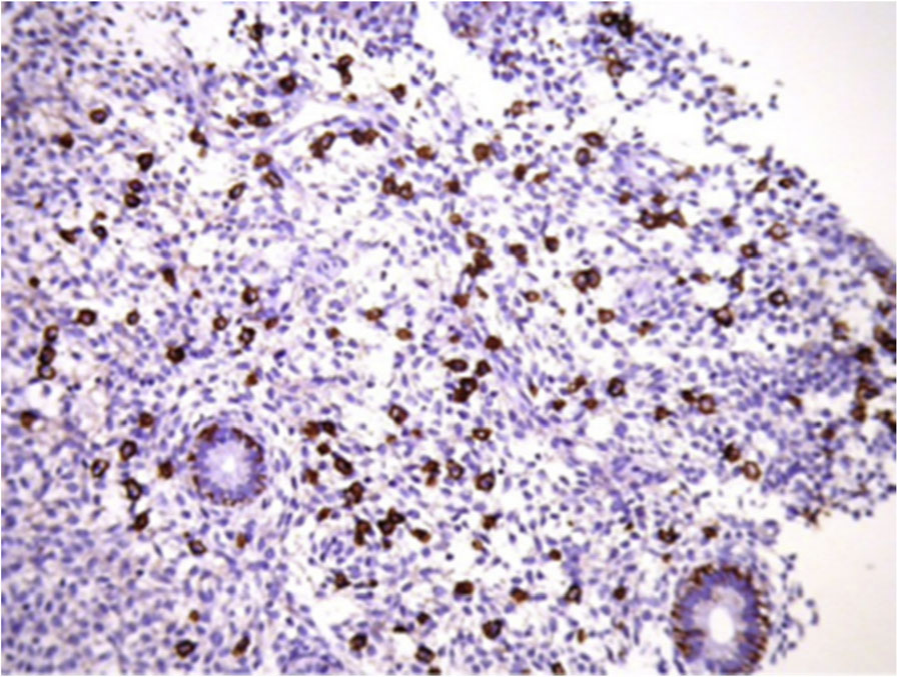
Plasma cell identification (brown color) with immunostaining for syndecan-1 (CD 138) in endometrial stroma

**Fig. 2:**
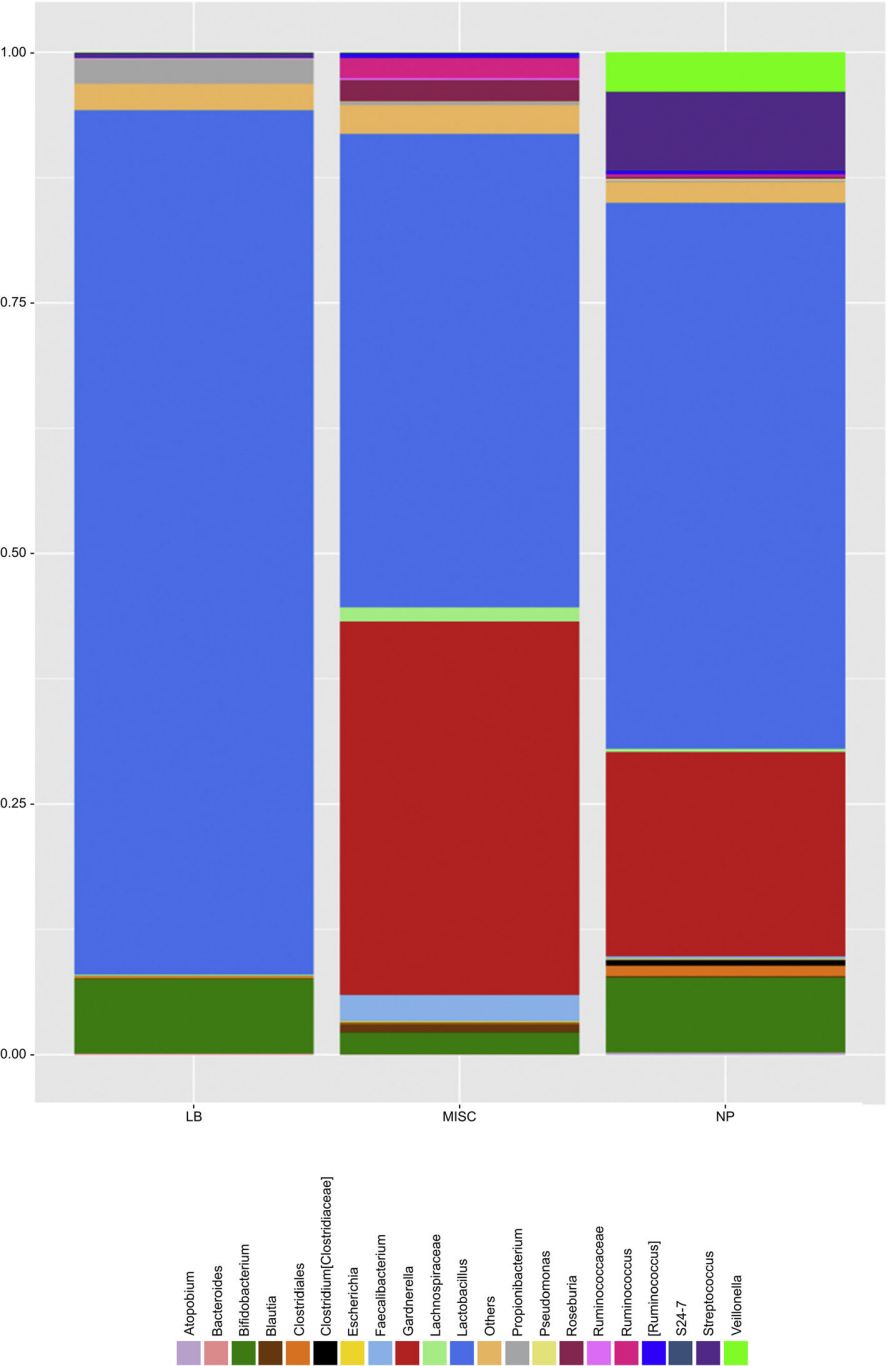
Low abundance of endometrial *Lactobacillus* is associated with poor reproductive outcome

Many women who have experienced RIF have also been determined to have chronic endometritis (CE) from bacterial colonization, often with minimal or no clinical signs of infection [44]. Kushnir et al. found that among a sample of infertile patients, 45% had CE, particularly those with RIF [45]. CE is a uterine pathology that has traditionally been diagnosed on histological examination, on visualization with hysteroscopy, and by bacterial culture. Immunohistochemistry stain for syndecan-1(CD 138), (Fig. 1) a plasma cell marker, can be used to provide a more accurate diagnosis, though agreement on a standard number of plasma cells present is not yet established. Bouet et al. confirmed a prevalence of 14% of CE using histological evaluation in RIF patients. In addition, hysteroscopy had a 40% sensitivity for detection of CE, visualizing mucosal edema, endometrial hyperemia, and the presence of micropolyps (all part of diagnostic criteria for CE) [46, 47]. Cicinelli et al. found that 66% of women were diagnosed with CE via hysteroscopy, 57.5% with histology, and 45% were also culture positive. Though culture is the least reliable method of detecting CE, it did allow for specific pathogens to be detected for targeted therapy. Most of the pathogens found consist of common bacteria like Group B *Streptococcus*, *Escheria Coli*, and *Enterococcus Faecalis*, or *Mycoplasma*. In some cases, sexually transmitted infections such as *Chlamydia* can be responsible [44]. A molecular method of diagnosis of chronic endometritis has shown promising results as presented by Moreno et al. in a new study published in June 2018. Real time polymerase chain reaction (RT-PCR) can identify bacterial DNA with an agreement of 76.92% when samples showed concordance by hysteroscopy, histology, and culture. RT-PCR showed 75% sensitivity and 100% specificity compared with the concordant results of the other three tests. It allows for detection of culturable and unculturable bacteria colonizing the endometrium even without histological signs of infection. This molecular test shows promise as a faster and more reliable tool for a streamlined diagnosis of chronic endometritis [48]. The bacteria present in the endometrium lead to abnormal lymphocyte counts and as a result, an environment that interrupts normal endometrial receptivity. Implantation rate of those cured of infection was 37%, compared with 17% in those that were not, however these rates did not reach statistical significance. (See treatment section for details on antibiotic protocols) However, live birth rates with next cycle of IVF after infection was cured was significant, with a rate of 61% in comparison with 13% in those that could not be cured with antibiotics [44]. In some cases, it is not necessarily chronic infection that leads to decreased implantation rates, but rather the constituents of bacterial flora present in the endometrium. While it was once thought that the endometrium was a sterile environment, it has now been accepted that *Lactobacillus* colonize this region in addition to the vagina. In a recent study, Moreno et al. demonstrated that women with *Lactobacillus* dominated endometrium undergoing IVF have been shown to achieve higher rates of successful implantation (60.7% vs. 23.1%) and live birth (58.8% vs. 6.75%) rates compared to those with non-Lactobacillus dominated endometrium (*Gardnerella*, *Streptococcus,* and other organisms present) (Fig. 2) [49].

**Fig. 3:**
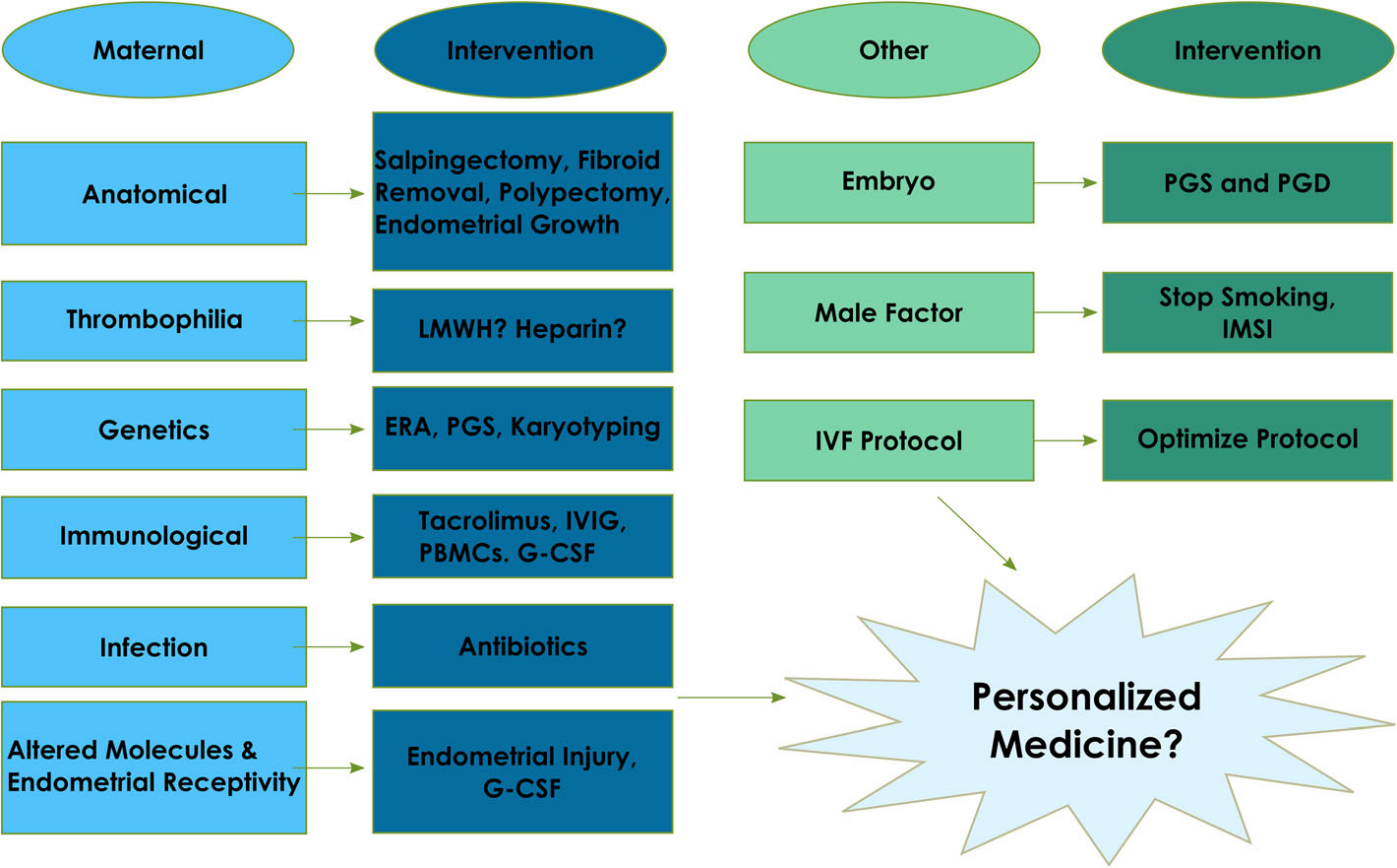
Summary of etiology and treatment options for RIF

### Altered expression of associated molecules

#### Hyperglycosylated hCG

Hyperglycosylated hCG (hhCG) is produced by cytotrophoblast cells and is thought to assist the embryo’s invasion into the decidua during implantation, which might make it a good marker of implantation [50]. It is also the main form of hCG produced in first 3 weeks of pregnancy [51]. This large hyperglycosylated hCG molecule closely resembles the structure of transforming growth factor beta (TGF- β) and binds to its receptor which promotes cell growth and inhibits apoptosis. Though the levels of total hCG were similar in women with biochemical pregnancy, early miscarriage, and live birth outcomes, mean concentrations of hyperglycosylated hCG in the urine have been shown to be significantly lower in women who had biochemical pregnancy (0.63 ± 1.3 mIU/ml) in comparison in those with successful term outcomes (5.4 ± 4.3 mIU/ml, *P* < .001) [50]. Bersinger et al. showed similar results in that, hyperglycosylated hCG was significantly decreased 16 fold (*P* < .0001) in biochemical pregnancy when compared with ongoing clinical pregnancies. Since most women undergoing IVF receive hCG to induce final follicular maturation, it takes several days to be cleared, and therefore the presence of hyperglycosylated hCG can serve as one of the earliest markers of pregnancy. An abnormally low level may alert the physician earlier of a potentially abnormal pregnancy [52]. Strom et al. determined that serum values of hhCG at 6 days after blastocyst embryo transfer could be used to diagnose clinical pregnancy (hhCG> 300 pg/ml), biochemical pregnancy (hhCG 75–300 pg/ml), and absence of pregnancy (hhCG < 75 pg/ml). Distinction between biochemical and clinical pregnancy by hhCG level had a specificity of 100% and sensitivity of 92% at day 6. It is not clear whether low hhCG levels are a cause or effect of the implantation failure [51]. Though more studies with larger samples need to be conducted to validate this data, this might be a potential test for a very early evaluation of pregnancy outcome. It is also relevant to address that Butler et al. found that in 9 of 15 home pregnancy tests, hhCG levels could not be detected as well as hCG levels even though hhCG is the principle molecule present in early pregnancy [53].

#### Leukaemia inhibitory factor (LIF)

Leukaemia inhibitory factor (LIF), part of the IL-6 family of cytokines, is one of the factors that has been explored in relation to endometrial receptivity. Low levels of LIF have been associated with higher risks of implantation failure [54]. Hambartsoumian demonstrated that LIF production is decreased in women with RIF. Women with RIF had decreased LIF in the secretory phase of the menstrual cycle when compared with the proliferative phase (2162 ± 541 pg vs. 4953 ± 1525 pg, *P* = .06). In contrast, there was no statistically significantly difference in LIF levels between the two phases of the menstrual cycle in controls [55].

#### Other molecules

There are several additional molecules which have been suggested to play a role in endometrial receptivity and implantation including prostaglandins (PG) and cellular adhesion molecules (CAMs). Phospholipase A_2_ (PLA_2_) is one of the key enzymes involved in the release of arachidonic acid, and cyclooxygenase enzyme 2 (COX-2) further downstream is one of the key enzymes involved in the production of PG synthesis. PGs have been suggested to play a role in reproductive processes such as ovulation and implantation, though the mechanism is not well understood. Achache et al. suggests that decreased PG synthesis has a role in RIF. Levels of cytosolic phospholipase A2 (cPLA_2a_) (1.001 vs. 2.956; *P* < .05) and COX-2 (0.195 vs. 0.959; *P* < .0085) were found to be decreased in patients with RIF, when compared with fertile controls, and it may be only detrimental to implantation when both enzymes are missing concurrently. In response to the decreased function and presence of these enzymes, secretory phospholipase A2 (sPLA2-IIA) was overexpressed as a compensatory pathway to maintain release of arachidonic acid [56].

CAMs, and in particular integrins which function in cell to cell interactions, are thought to play a substantial role in implantation. Specifically αvβ3, α4β1, and α1β1 are expressed during the implantation window. Thomas et al. studied this by taking endometrial biopsies of women, adding antibodies to these integrins, and then staining for their presence in the glandular and luminal epithelium. There was a statistically significant greater expression of αvβ3 in the luminal epithelium in those who became pregnant in their next IVF cycle than in those that did not (1.6 (1.1–1.9) vs. 1.1 (0.8–1.6), *P* < .027), expressed here by HSCORE. There was also a statistically greater expression of α4β1in the glandular epithelium in those who did not become pregnant in their next IVF cycle (2.7 (2.1–3.3) vs. 2.3 (1.5–2.8) vs *P* < .047). Lack of αvβ3 expression is often seen with delayed histological development or out of phase endometrium, and it is important to continue to evaluate the role of this integrin expression in implantation [57]. Table 1 summarizes the molecules suggested to play a role in implantation failure.

**Table 1:** Summary of molecules suggested to play a role in implantation failure

Molecule	Source	Implantation Failure, Biochemical Pregnancy, and RIF patients	Controls/healthy outcomes	Source
Hyperglycosylated hCG (levels)	Urine	0.63 ± 1.3 mIU/ml	5.4 ± 4.3 mIU/ml	*Cole* et al*, 2012* [50]
		Biochemical Pregnancy	Healthy Delivery	
		(*P* < .0001)	(*P* < .0001)	
Prostaglandins (cPLA_2a_)	Endometrium	1.001 (ratio)	2.956 (ratio)	*Achache* et al.*, 2010* [56]
		RIF patients	Controls	
		(*P* < .05)	(*P* < .05)	
LIF (in secretory phase of menstrual cycle compared with proliferative phase)	Endometrium	Secretory 2162 ± 541 pg	Secretory 3489 ± 967 pg	*Hambartsoumian* *1998* [55]
		Proliferative 4953 ± 1525 pg	Proliferative 4698 ± 1136 pg	
		(*P* = .06)	NS	
		RIF Patients	Controls	
Cellular adhesion molecules 1) αvβ3 luminal epithelium α4β1 glandular epithelium	Endometrium	HSCORE	HSCORE	*Thomas* et al.*, 2002* [57]
		1.1 (0.8–1.6)	1.6 (1.1–1.9)	
		no pregnancy	pregnancy	
		(*P* = .027)	(*P* = .027)	
		2.7 (2.1–3.3)	2.3 (1.5–2.8)	
		no pregnancy	pregnancy	
		(*P* < .047)	(*P* < .047)

**Table 2:** Summary of results of treatment options with regard to implantation rate, clinical pregnancy rate, and live birth rate

	Implantation Rate	Clinical Pregnancy Rate	Live Birth Outcome	Source
	(treatment vs. control)	(treatment vs. control)	(treatment vs. control)	
Tacrolimus	45.7% vs. 0%	64% vs. 0%	60% vs. 0%	*Nakagawa* et al.*, 2015* [36]
	(*P* < 0.0001)	(*P* < 0.0001)	(*P* < 0.0001)	
IVIG	34.4% vs. 13.7%	60.2% vs. 39.3%	49.8% vs. 31.6%	*Li* et al.*, 2013* [82] *(meta-analysis)*
	RR 2.708	RR 1.475	RR 1.616	
	(95% CI: 1.302–5.629, I2 = 65.0%)	(95% CI: 1.191–1.825, I2 = 65.7%)	(95% CI: 1.243–2.101, I2 = 58.2%)	
PBMC	22% vs. 4.88%	39.58% vs. 14.29%	33.33% vs.9.58%	*Li* et al.*, 2017* [84]
	(*P* = 0.014)	(*P* = 0.038)	(*P* = 0.038)	
G-CSF	31.5% vs. 13.9%	48.1% vs. 25%	33.3% vs. 17.3%	*Xu* et al.*, 2015* [87]
	(*P* < 0.01)	(*P* < 0.01)	NS	
Antibiotics for CE	37% vs. 17%	65.2% vs. 33%	60.8% vs. 13.3%	*Cicinelli* et al.*, 2015* [44]
	NS	(*P* = 0.039)	(*P* = 0.02)	
Salpingectomy	25.6% vs. 12.3%	45.7% vs. 22.5%	40% vs. 17.5%	*Strandell* et al.*, 1999* [94]
	(*P* = 0.038)	(*P* = 0.029)	(*P* = 0.038)	
Endometrial Biopsy	27.7%, vs. 14.2%	66.7% vs. 30.3%	48.9% vs. 22.5%	*Barash* et al.*, 2003* [96]
	(*P* = .00011)	(*P* = .00009)	(*P* = .016)	
IMSI procedure	19.2% vs. 7.8%	43.1% vs. 10.5%	34.7% vs. 0%	*Shalom-Paz* et al.*, 2015* [109]
	(*P* = 0.042)	(*P* = 0.02)	(*P* = 0.003)	

### Anatomical abnormalities and endometrial thickness

There are several types of uterine pathologies including polyps, myomas, and adhesions that can impact implantation rates in patients undergoing IVF. Most of the time the patients are asymptomatic and sometimes these problems can even go unnoticed on transvaginal ultrasound suggesting that another method of uterine cavity assesssment such as hysteroscopy be required (see treatment section for more details) [58]. Myomas can cause a distortion of the endometrial cavity and adhesions which often develop following surgery or infection can prevent attachment of the embryo to the luminal surface [4]. The ASRM has reported that hydrosalpinges can also have a negative impact on implantation in women undergoing IVF. Though not well understood, several mechanisms have been considered such as deprivation of proper embryo development, due to the fluid’s lack of nutrients and energy [59]. In addition, the fluid can negatively impact endometrial receptivity, and can also physically flush the embryo out preventing implantation [60]. Mullerian abnormalities such as septate uterus and bicornuate uterus should also be taken into consideration in patients with RIF. Septate uterus might contribute to RIF as those with untreated septa have poor outcomes after IVF in comparison with those who underwent hysteroscopic metroplasty. However, women with a bicornuate uterus usually have normal implantation, but these patients have a higher risk of mid trimester pregnancy loss [4].

The endometrium itself can also be the source of implantation failure. Previous endometrial trauma, prolonged use of birth control pills, and impaired uterine blood flow are all potential etiologies of a thin endometrium [61]. Evaluating the blood flow in the uterine radial arteries is useful in assessing the degree of blood flow to the endometrium. When there is a decrease in blood flow, epithelial cell growth and vascular endothelial growth factor (VEGF) production can be reduced, which ultimately deprives the endometrium of necessary angiogenesis and growth factors to reach proper thickness for successful implantation. Miwa et al. conducted a transvaginal Doppler ultrasonography study that found that uterine radial artery resistance index was significantly higher in patients with thin endometrium < 8 mm than with normal thickness endometrium > 8 mm (0.852 (0.826–0.955) vs. 0.751 (0.549–0.840), *P* < .05) [62] The thickness of the endometrium has been determined to have an effect on implantation rates. Though an exact threshold for proper endometrial thickness has not been unanimously agreed upon, it is suggested that about 8 mm is the lower limit for which ART can still usually be successful. From a thickness of 9 mm to 16 mm chance of pregnancy increases from 53 to 77% showing that a significant difference does exist in implantation rates [63].

### Genetics

Chromosomal abnormalities including translocations, mosaicism, inversions, and deletions are more frequent in RIF patients than the general population, and yet the prevalence is only about 2%. The most common abnormality is translocation [64, 65]. Voullaire et al. looked at the difference between women with aneuploidy in one or two chromosomes, and women with complex abnormalities, defined as three or more chromosomes with aneuploidy. Though this is a rare occurrence, it was shown to be significantly more likely to be in women who had RIF, whereas aneuploidy in only one or two chromosomes was not considered to be a significant cause of RIF. It is accepted that this complex abnormality is mitotically derived as it is observed more commonly in the embryos than in the oocytes upon collection, though it is unclear what exactly causes this problem. [66] It is recommended that parental karyotyping is involved in cases of women suffering from RIF, but only in specific subgroups such as nulliparous women with history of miscarriage, as it would be very expensive to make it a universal test, and it would only protect a small group of patients. In addition, men with severe infertility are also recommended for karyotyping [64]. In 2016 Koot et al. published a study which found an endometrial gene signature made up of 303 genes which was found to be predictive of RIF. RIF patients had down regulation of genes that were involved in cell regulation and division and cytoskeleton and cilia formation. It is interesting to note that there was one group of RIF patients who were consistently incorrectly classified with a > 90% error rate, suggesting that this is not random, but that the underlying etiology in these patients is not due to the expression of this group of endometrial genes, further confirming the complexity of the approach to evaluation and treatment of RIF [67].

## Therapeutic interventions for recurrent implantation failure

### Optimal IVF treatment

#### Embryo factors

There is ongoing debate about optimal conditions for successful IVF outcomes. Currently the implantation rate per embryo is only about 15% [5]. Many IVF clinics in the past decade have moved towards using blastocysts for embryo transfer instead of cleavage stage embryos. Guerif et al. found that the quality of the embryo used for transfer was important for implantation success, regarding both blastocysts (day 5/6) and cleavage stage embryos (day 2) in patients with RIF. However, there were higher implantation rates in the blastocyst group (25.4%) compared with the cleavage stage embryo group (12.4%) despite embryo quality [68]. Levitas et al. found that in patients with RIF with good ovarian response during IVF the implantation rates were greater in patients undergoing blastocyst transfer (21.2%) than patients undergoing cleavage stage embryo transfer (6%). In addition, fewer embryos were actually transferred per cycle in the blastocyst group (3.4 ± 0.7 vs. 1.9 ± 0.4) [69]. It is also relevant to note that according to Gleicher et al. it seems that some of the literature indicating this success might be misleading since some of the conclusions have been generalized to populations not represented by the study patient population. They suggest that all IVF studies should clearly describe the patient population treated including age and functional ovarian reserve, and that they cannot unanimously be applied to all IVF patients, many of whom might not meet the parameters of the study [70]. A 2016 Cochrane review determined that evidence of blastocyst transfer over cleavage stage embryo transfer was of low quality for live birth outcome, and only moderate quality for clinical pregnancy outcome [71].

In addition to embryo stage, other parameters of IVF protocols have also been evaluated. There is still a debate about whether frozen embryo transfer or fresh embryo transfer leads to better outcomes, though frozen embryo transfer is becoming the more popular choice. Shapiro et al. conducted a study in 2011 that determined that implantation rate (70.8% vs. 38.9%, *P* < .0001.), clinical pregnancy rate (84% vs. 54.7%, *P* = .0013) and ongoing pregnancy rate (78% vs. 50.9%, *P* = .0072) were all significantly higher in those undergoing frozen embryo transfer compared with those using fresh embryo transfer. This evidence supports the idea that there is impaired endometrial receptivity right after ovarian stimulation seen with fresh transfers, whereas frozen cycles have artificial endometrial preparations [72]. The Society for Assisted Reproductive Technology (SART) reported that from 2006 to 2012 there was an 82.5% increase in using FET and only a 3.1% increase in using fresh cycle starts, suggesting a strong trend toward FET use [73]. A Finnish cohort study conducted between 1995 and 2006 showed that there were benefits to the neonate such as decreased risks of preterm birth, low birth weight, and gestational age size in the frozen embryo transfer group compared with the fresh embryo transfer group. Many benefits of frozen embryo transfer also include the fact that hormones are given at levels to mimic natural cycles since ovarian stimulation and oocyte retrieval were done prior, and that better quality embryos are more likely to survive the freezing and thawing process [74]. In an attempt to settle this debate a randomized control trial was published in the New England Journal of Medicine in early 2018 to assess the live birth outcomes of women receiving frozen embryo transfer compared with fresh embryo transfer. The study demonstrated that there was no significant difference in outcome in live birth rate between women who underwent frozen embryo and fresh embryo transfer with cleavage stage embryos (48.7% vs. 50.2% RR, 0.97; 95% CI, 0.89 to 1.06; *P* = .5). These results may not be applicable to those undergoing blastocyst transfer due to differences in the embryo-endometrial cross talk at the different stages of the embryo development and endometrial preparation. In addition, there was no significant difference in biochemical pregnancy, implantation, clinical pregnancy, or neonatal outcomes between the two groups. The only significant difference between the methods was that frozen embryo transfer resulted in lower rates of ovarian hyperstimulation syndrome (.6% vs. 2% RR, 0.32; 95% CI 0.14 to 0.74; *P* = .005) which has also been reported in other studies [75].

#### Transfer methods

There are several different types of methods used for embryo transfer which can impact implantation and pregnancy outcome. Ultrasound guided transfer led to higher rates of clinical pregnancy and live births. The ideal type of catheter used (rigid versus soft) can be dependent on cervical shape. Additionally, in some cases removing cervical mucus via aspiration can lead to more successful pregnancy outcome [4].

#### Ovulation induction protocol

In addition to embryo type and transfer, the use of different controlled ovarian stimulation protocols is a topic of discussion. The effects of GnRH agonists versus antagonists in IVF protocol on implantation rates have been evaluated both on their own and when used in conjunction with oral contraceptive pre-treatment. A meta-analysis was published in early 2017 showing that in the general IVF population the group treated with antagonists had lower pregnancy rates than those treated with agonists (RR 0.89, 95% CI 0.82–0.96). However, those treated with antagonists had lower rates of ovarian hyperstimulation syndrome (RR 0.63, 95% CI 0.50–0.81), a complication of IVF [76]. Barmat et al. found no significant differences in implantation rates, and the only major difference found was the convenience of patient oocyte retrieval in the group using the GnRH antagonist due to shorter timing of protocol [77]. Orvieto et al. recently suggested a new protocol for IVF in patients with RIF which includes using both GnRH-agonists and antagonists with administration of GnRH agonist and hCG double trigger for final follicular maturation prior to oocyte retrieval. Embryos were frozen and then transferred after endometrial injury and hysteroscopy in a natural cycle with modified luteal support, including progesterone supplementation, and hCG and GnRH agonists were injected at that time and 4 days following Day-3 embryo transfer respectively [5]. This observation calls for larger trials and also implicates that IVF protocols have a significant impact on oocyte embryo quality and endometrial receptivity. It will be important to determine if a specific stimulation protocol for IVF in RIF patients will make a significant difference in implantation and birth outcomes. However, this could also differ in success rate depending on the etiology of patient’s implantation failure as well as other important clinical parameters including maternal age.

#### Progesterone support

It is well known that progesterone support is a significant part of IVF protocols. As in RPL, progesterone type may play an important part in increasing the birth rate in RIF patients. A systematic review and meta analysis by Saccone et al. showed a clear benefit of using progesterone in early pregnancy for women suffering from recurrent pregnancy loss. More specifically, dydrogesterone was found to be superior to other types of progesterone [78]. A very recent meta analysis and systematic review performed by Roepke et al. in 2018 concluded that according to evidence based medicine, the literature does not advise on any specific treatment for idiopathic recurrent pregnancy loss, with the exception of progesterone administration starting from ovulation [79]. In addition, a randomized control trial conducted by Tournaye et al. found oral dydrogesterone to be non inferior to vaginal progesterone for luteal support among patients undergoing IVF. There was no significant difference in clinical pregnancy rate at 12 weeks gestation among the two groups. Oral dydrogesterone has fewer side effects than vaginal gestation among the two groups. Oral dydrogesterone has fewer side effects than vaginal progesterone, and is administered orally making it easier for patient adherence [80]. Thus, progesterone and specifically orally administered dydrogesterone used in IVF protocols, may have a significant role in improving pregnancy and live birth rates among patients with RIF mainly when started in the luteal phase. More studies are needed to support this.

### Antithrombotic agents

Heparin has been evaluated for use in RIF patients, though there is not yet evidence to recommend its use for improved pregnancy outcomes in these patients. A group of RIF patients treated with low molecular weight heparin had almost identical implantation, clinical pregnancy, and live birth rate outcomes when compared with controls [81].

### Immunotherapy

Several different immunological therapies used to increase rates of implantation have been studied such as Tacrolimus, intravenous immunoglobulin (IVIG), peripheral blood mononuclear cells, and granulocyte colony stimulating factor. An elevated Th1/Th2 ratio has been shown to negatively impact implantation rates. However, IFNg is a Th1 specific cytokine that is necessary in some degree in arterial development for implantation, and yet levels beyond a certain threshold are implicated in implantation failure.

#### Tacrolimus

Tacrolimus is an immunosuppressive drug currently approved for immunological allograft transplant rejection. It has been used by Nakagawa et al. as a plausible treatment for RIF patients with an elevated Th1/Th2 ratio. However, due to the delicate balance of cytokine levels that must be maintained, dosing must be specifically adjusted in order to maintain certain levels of Th1 cytokines essential to the process of implantation. The women with elevated ratios treated with Tacrolimus achieved 45.7% success rate with implantation, whereas those without treatment had 0% successful implantations (*P* < 0.0001). Those treated with Tacrolimus also achieved a 60% live birthrate, whereas those in the control group had 0% live birth rate (*P* < 0.0001) [36]. This indicates that this immunological imbalance might play a significant role in some patients with recurrent implantation failure.

#### Intravenous immunoglobulin

IVIG treatment has also been regarded as a possible immunological therapy for women suffering from repeated implantation failure with an elevated Th1/TH2 ratio, elevated NK cells, an abnormal TNFa/IL-10 ratio, and auto-antibodies. In a meta-analysis conducted by Li et al., the effect of IVIG administration on implantation rate was examined across six studies. Implantation rates were 34.3% in patients given IVIG, and only 13.7% in patients receiving no treatment or a placebo, with a relative risk of 2.708 (95% CI, 1.302–5.629). Clinical pregnancy occurred in 60.2% of participants treated with IVIG, and in 39.3% of those either treated with a placebo or not treated. The use of IVIG was associated with a significantly higher clinical pregnancy rate and the RR was 1.475 (95% CI: 1.191–1.825). Live birth rate outcomes in patients given IVIG were 49.8% in comparison with 31.6% in the placebo group, with a relative risk of 1.616 (95% CI, 1.243–2.101) [82].

#### Peripheral blood mononuclear cells

Peripheral blood mononuclear cells (PBMCs) consist of B lymphocytes, T lymphocytes, and monocytes. These cells produce many cytokines that have been known to improve endometrial receptivity during implantation. PBMC injection via intrauterine insemination catheter before embryo transfer greatly improved the outcome of implantation in those who previously suffered from RIF. Yu et al. confirmed that implantation rate was significantly higher in the group treated with intrauterine administration of PBMCs than the control group (23.66% vs. 11.43%) [83]. Li et al. found that only patients with four or more previous implantation failures could benefit from PBMC administration. In these patients there was a significant increase in implantation rate (22% vs. 4.88%, *P* = 0.014), clinical pregnancy rate (39.58% vs. 14.29%, *P* = 0.038), and live birth rate per embryo transfer cycle (33.33% vs. 9.58%, *P* = 0.038) after their next frozen embryo transfer [84].

#### Granulocyte Colony stimulating factor

Gleicher et al. conducted one of the first small studies on use of granulocyte colony stimulating factor (G-CSF) as a treatment of thin endometrium on four women. All four women had an initial endometrial thickness of 3–6.5 mm, and with this intervention all had an endometrial thickness > 7 mm when they had their embryo transfer. All four women actually had successful implantation, though it is important to keep in mind the small sample size [85]. Gleicher conducted a follow up study with a larger sample and found similar results in favor of G-CSF to increase endometrial thickness. Thickness of the endometrium was increased within 48 h [86]. Xu et al. also found a significantly higher implantation rate in those treated with G-CSF in comparison with controls (31.5% versus 13. 9%, *P* < .01) and a significantly higher clinical pregnancy rate (48.1% vs. 25%, *P* < .01). In contrast, there was a higher live birth rate compared with the controls it was not statistically significant (33.3% vs. 17.3%) [87]. Li et al. conducted a meta-analysis which determined that G-CSF use in women with thin endometrium or with repeated IVF failure had a higher implantation rate, however, this did not reach statistical significance in the general population (RR 1.461; 95% CI: 0.801, 2.664, I^2^ = 70.7%) as it did in the Asian population (RR = 1.887; 95% CI: 1.256, 2.833, I^2^ = 23.2%). In contrast, the use of G-CSF when compared with no treatment or placebos was associated with significantly higher biochemical (RR 2.385 95% CI:1.414, 4.023, I^2^ = 0.0%) and clinical pregnancy rates (RR 2.312, 95% CI: 1.444, 3.701, I^2^ = 0.0%) among women with thin endometrium or repeated IVF failures in the general population. This suggests that G-CSF might be an important factor in improving implantation outcomes in specific populations of women suffering from RIF [88]. Additional treatments that have been implemented for thin endometrium include Vitamin E to improve blood flow leading to increased endometrial proliferation [62], and sildenafil citrate suppositories which reported by Sher and Fisch also improved blood flow, growth, and pregnancy rate in 70% of patients undergoing IVF [89].

### Antibiotics for infection

In patients who have had CE diagnosed via hysteroscopy and culture, antibiotic therapy has been shown to be an effective intervention to cure most infections, leading to more successful implantation rates in future IVF cycles. Women infected with common gram positive bacteria, *Enterococcus* and *Strep Agalactiae*, were given Amoxicillin and Clavulanate twice a day for 8 days, and gram negative bacteria such as *Escheria Coli* were given ciprofloxacin twice a day for 10 days*.* Women with Mycoplasma and Ureaplasma were treated with 1g Josamycin twice a day for 12 days with the addition of Minocycline in persistent cases. When infections were cured, the implantation rate was found to be higher in the next cycle at 37%, though not statistically significant in comparison with a rate of 17% in those who had persistent infection even after three antibiotic treatments. The clinical pregnancy rate in those with CE who cleared their infection with antibiotics was 65.2% in comparison with 33% in those with persistent infection (*P* = 0.039). The live birth rate in those who had cleared their CE with antibiotics was 60.8%, significantly higher than the 13.3% in those who had not cleared the infection (*P* = 0.02) [44]. There is currently a clinical trial at the University of Valencia in Spain recruiting participants aiming to find a new non-invasive diagnostic test for CE, using bacterial DNA retrieved from endometrial fluid. The antibiotics given to patients who test positive for CE will be determined based on bacteria detected on sequencing and quantitative PCR. The new sequencing technique will be compared with patients who have either biopsy or culture to determine if the new technology has greater efficacy [90]. There is another trial being conducted at Fu Xing hospital in Beijing China which is evaluating whether the use of amoxicillin and clavulanate will cure the CE and ultimately lead to greater live birth rates. This trial will evaluate the presence of CE solely using hysteroscopy and immunohistochemical staining of CD 138 antibody (Syndecan 1) [91].

### Anatomical intervention

#### Correction of intra-uterine pathologies

Polyps, myomas, adhesions, and septa can all affect implantation, and the gold standard for evaluation is hysteroscopy. The previously reported prevalence of undetected anomalies was between 20 and 45%, however, Fatemi et al. found the prevalence in their study population to be only 11%, identifying polyps as the most common pathology (41%) [92]. Cenksoy et al. demonstrated dramatically different findings, in that 44.9% of patients in their study had abnormal hysteroscopic results. After corrected pathology, 51% of these women became pregnant. The implantation rate was significantly higher in those who had corrected polyps (*P* = .001), but not those with corrected adhesions [58], suggesting that different pathologies may not have the same successful implantation and pregnancy rate after intervention. Demirol et al. found that clinical pregnancy rates were significantly higher in those that had hysteroscopy and treatment for polyps and adhesions in comparison with those who did not have hysteroscopic evaluation (30.4% vs. 21.6%, *P* < .05). Many of the patients with abnormal hysteroscopy findings had normal hysterosalpingogram results [93]. Hysteroscopy might serve as a useful diagnostic tool in many RIF patients, as some literature suggests that with this intervention there can be major changes in pregnancy outcome.

#### Salpingectomy

Salpingectomy and in some cases tubal occlusion procedures in the presence of hydrosalpinges have been shown to increase the likelihood of implantation success in future IVF cycles [60]. Strandell et al. found that implantation rates as well as clinical pregnancy rates, and delivery rates in patients with bilateral hydrosalpinges who were treated with salpingectomy, increased significantly in their next IVF cycle compared with those who did not undergo the procedure. Implantation rates were 25.6% in those who underwent salpingectomy compared with 12.3% in those that did not have the procedure (*P* = 0.038). Clinical Pregnancy rates were 45.7% in those with salpingectomy in comparison to 22.5% with those without it (*P* = 0.029). Live birth rates were 40% in those who had salpingectomy compared with 17.5% in the control group (*P* = 0.038) [94]. Kontoravdis et al. had similar findings that implantation rates (24.8% vs. 5.6%), clinical pregnancy rates (55.3% vs. 14.3%), and ongoing pregnancy rates (48.9% vs. 7.1%) were significantly higher in those who underwent salpingectomy than those who did not have treatment [95]. Seli et al. found that although levels of LIF were significantly lower in their patient group with hydrosalpinges than their fertile controls, after salpingectomy normal levels of LIF were measured and pregnancy rates increased in these patients by 231 ± 49% (mean ± SEM) [54].

#### Endometrial injury (biopsy)

Many studies suggest that injury to the endometrium prior to implantation in IVF patients causes decidualization, preparing the endometrium for implantation. One mechanism by which this may occur is by an increase in local cytokines involved in wound healing such as LIF and IL-11, both important in the implantation process [6]. Barash et al. were the first to report on the topic, that the implantation rate of the patients who underwent biopsy (injury) prior to their IVF cycle was 27.7%, significantly higher than the 14.2% implantation rate in those that did not undergo any endometrial scratching (*P* = .00011). Clinical pregnancy rate was significantly higher in the group that had endometrial biopsy than the control group (66.7% vs. 30.3%, *P* = 0.00009). In addition, the live birth rate was 48.9% in those that had endometrial biopsy, which was significantly higher than 22.5% in the control group (*P* = 0.016) [96]. Gibreel et al. found both a significantly higher biochemical pregnancy rate (29.6% vs. 11.7%) and clinical pregnancy rate (25.9% vs. 9.8%) in patients undergoing endometrial biopsy, than those undergoing a placebo procedure. Both groups of women were given doxycycline after the procedure to prevent possible infection [97]. Siristatidis et al. published a study in 2016 that showed that clinical pregnancy rate and live birth rate in patients with RIF were both higher in the group that underwent hysteroscopy and endometrial cutting than in the control group (39.2% vs. 23.1% and 35.3% vs. 15.4% respectively) Miscarriage rates and biochemical pregnancy rates were not statistically different between the groups [98]. This simple procedure with no side effects may eventually lead to reduction in the number of IVF attempts needed to achieve successful implantation. However, there is no agreement on what degree of injury, the number of injuries, or when in the menstrual cycle this procedure must occur to work, if at all. This is a widely used treatment, though there is insufficient evidence demonstrating a strict protocol for how to perform this procedure, and therefore more data is needed [99]. A 2015 Cochrane review calls for more trials suggesting that there is only moderate quality evidence that endometrial injury done between day 7 of the previous cycle and day 7 of the embryo transfer cycle can lead to increased clinical pregnancy and live birth rates in women with previous embryo transfer [100].

### Genetics

#### Pre-implantation genetic screening

Pre-implantation genetic screening (PGS) was developed in response to the high number of chromosomal abnormalities seen in patients with spontaneous miscarriage. Though initially it was hypothesized that PGS might be helpful in improving pregnancy outcomes in patients with RIF recent studies such as that conducted by Rubio et al. demonstrated that there was no significant difference in implantation rate (36.6% vs, 21.4%), clinical pregnancy rate (53.5% vs. 33.3%), or live birth rate (47.% vs. 27.9%) between those evaluated and not evaluated with PGS [101]. Hatirnaz et al. published a study in 2017 which also determined that PGS had no significant outcome on clinical pregnancy and live birth rates in patients with recurrent IVF failure. It has been hypothesized that this might be due to mosaicism in abnormal embryos. It is possible that the trophectoderm biopsy from these embryos is not representative of the chromosomal make up of all of the embryo’s cells [102]. In addition, Greco et al. found that implanted mosaic embryos can actually develop into healthy newborns with normal genetic makeup, though this was shown in a small sample size [103]. This suggests that PGS could actually misinform couples about the possible outcomes of their pregnancies. However, a 2016 study by Coulam et al. determined that women over the age of 40 years old did have a significant increase in pregnancy rates, measured by fetal cardiac activity, after a cumulative of four embryos were transferred (*P* = 0.03). While PGS does help select best quality embryo for transfer, the more embryos that are transferred with consistent rates of failed pregnancy, the less likely it is that the embryo is the problem in these cases [104].

#### Pre-implantation genetic diagnosis

There is data that suggests that patients with RIF have more chromosome abnormalities within their embryos. As a result, pre-implantation genetic diagnosis (PGD) can be used to evaluate specific chromosomal diseases among IVF patients with specific risk factors. An earlier study by Pehlivan et al. found that there was a significantly higher rate of chromosomal anomalies, specifically aneuploidies among those with repeated implantation failure. In addition, when these patients had genetic screening of the embryos using FISH, and their normal embryos were selected for transfer, the implantation rate was 24.6%, comparable with young fertile controls, 24.1%. It is important to note that this was highly dependent on age, and these results applied only to those < 37 years old. In comparison in women > 37 years old their implantation rate was only 12.2%. Those women > 37 years old also had the highest rates of chromosomal abnormalities in chromosome 21 and 22, and those < 37 years old had higher rates of abnormalities in chromosome 13 [105]. This suggests that there may be additional mechanisms at play in those with advanced maternal age that impede successful implantation. It is recommended that women with RIF should be karyotyped to determine if they may have balanced translocations. PGD is recommended only in patients that have these balanced translocations, as these can lead to aneuploidy in their gametes [7].

#### Endometrial receptivity array

Implantation relies on cross talk between the embryo and the endometrium, with facilitation by many different factors such as growth factors, cytokines, cell adhesion molecules, and transcription factors. The implantation window, which exists when the optimal environment of these factors are balanced, usually lasts for only four or five days and begins around six days after ovulation [106]. One of the possible mechanisms involved in RIF is the change in endometrial receptivity. One of changes in receptivity might involve the shift in timing of the window of implantation (WOI), previously assumed to be the same among all women. Regulation and dysregulation of many different genes are implicated in the changes among the endometrium WOI. Ruiz-Alonso et al. used the endometrial receptivity array (ERA) test to identify window of implantation changes based on 238 genes among women with RIF. In 25% of patients the window of implantation was shifted, and once their embryo transfer time was changed based on personal data gathered from the ERA, the rates of implantation climbed to the rates of those with receptive endometrium with normal WOI. The mean age of patients in the groups in this study were 38.4 ± 4.7 in the RIF group and 39.1 ± 5.1 in the control group. However, 34.9% of the RIF group had oocyte donation, and 59.1% of the control group received oocyte donation. As a result, biological age of the embryos did not necessarily correlate with mother’s age [107]. A similar study was conducted by Hashimoto et al. in Japan. Using the ERA test, they determined whether the endometrium was receptive or non-receptive before performing embryo transfer. Implantation rate in the receptive group was not significantly different from the non-receptive group (32.8% vs. 31.6%). However, although the population in this study had a similar overall mean age, 38.42 ± 3.40 in the receptive group, and 40.08 ± 5.16 in the non-receptive group, there was only one case of oocyte donation [108]. This indicates that RIF origin in some patients might not actually be due to a pathological condition but rather require personalized evaluation to find ideal implantation time.

### Male factor

In addition to maternal factors playing a role in RIF, male factor, particularly spermatozoal morphology also can play a part. Spermatozoa have to have smooth nuclei with normal chromatin content and normal head shape to function properly. Intracytoplasmic morphologically selected sperm injection (IMSI) requires examination of spermatozoa under ultra-magnification X3600 before injection into the oocyte. While implantation rate (19.2% vs. 7.8%, *P* = 0.042), clinical pregnancy rate (43.1% vs. 10.5%, *P* = 0.02) and live birth rate (34.7% vs. 0%, *P* = 0.003) were reported to be significantly higher among those undergoing sperm selection via IMSI procedure than those undergoing simple intracytoplasmic sperm injection (ICSI) in a retrospective study conducted by Shalom-Paz et al. in 2015 [109], other studies could not demonstrate any benefit of using IMSI. There is still a lack of specific microscopic criteria for the assessment of sperm morphology, and therefore more studies are required to confirm the advantages of IMSI before a standardized clinical protocol can be created for this particular procedure [110]. A Cochrane review in 2013 called for further trials to be conducted since higher quality evidence needs to be established in order to recommend this technique for clinical practice [111]. Table 2 summarizes the results of treatment options with regard to implantation, clinical pregnancy, and live birth rates.

### Lifestyle modifications

In November 2017 The European Society of Human Reproduction and Embryology released a new set of guidelines which included recommendations for lifestyle modifications for RPL patients. Although RPL is a distinctly different disorder, there are similarities and many overlapping risk factors with RIF. Recommendations for RPL patients include smoking cessation because of its possible negative impact on live birth, and acheiving a healthy range BMI since obesity is associated with obstetric complications and lower chances of live birth. Stress has also been shown to be associated with RPL, but there is no evidence of a cause and effect relationship [2]. Evidence suggests that smoking, obesity, and high cortisol levels also play a role in implantation failure. Though more research needs to be conducted regarding the specific physiology behind these issues, lifestyle interventions such as assistance in quitting smoking, healthier diet and regular exercise, and emphasis on taking care of mental health may positively impact those suffering from RIF. These modifications require less invasive medical assistance and seem like an optimal first step in trying to change future implantation outcome in IVF treatment in couples struggling to get pregnant and have a successful delivery outcome. In addition, other behavioral changes may also be relevant to improve outcomes in these patients.

### Supportive treatment

A groundbreaking study published by Tamar Ben-Shaanan et al. in 2016 has led us to consider a completely new direction for RIF treatment intervention in addition to the previously mentioned therapies. It has long been suggested that the placebo effect has an association with activation of the reward system, yet the mechanism has not been known. This group demonstrated in a mouse model that the ventral tegmental area, one of the major components of the brain’s reward system and a dynamic player in the placebo response, when activated by positive experience, led to activation of the immune system. Both the innate and adaptive immune system were activated in response to exposure and re-exposure to *Escherichia coli* Bacteria. There was increased macrophage activity, decreased bacterial load, and greater lymphocyte response [112]. Since many etiologies of RIF are related to the immune system’s function, it seems that this could very well be applied to RIF patients. It may be a great first step in a new protocol for IVF patients to focus on creating an environment that would lead to activation of this reward system in couples suffering from RIF. Encouraging the patients to engage in activities that create enjoyment, and establishing a positive environment could be the first step in protocol for treating RIF patients. This intervention is relatively simple in that it does not involve medications or any invasive procedures. It can be done in the comfort of the patient’s familial and social circle, and on the patient’s own time. For each patient a positive activity or setting might mean something different, like spending 3 days meeting with friends each week, eating out in a nice restaurant once a week, making specific time for creative activity, or watching a comedy film a few times a week. This type of therapy can easily be tailored to each patient depending on personal hobbies and interests. This concept might be a great starting point for future studies in RIF treatment, and even now might be a great first step in protocol for evaluating and treating patients suffering from RIF.

## Conclusion

Recurrent Implantation Failure is a complex problem with a wide variety of etiologies and mechanisms as well as treatment options. (Fig. 3) The recommendations for women with RIF vary depending on the source of their problem. Perhaps the best and yet most complex answer is personalized medicine, a personal approach to each patient depending on her unique set of characteristics. There is not just one treatment option, but many depending on the etiology of the problem. However, it would help to establish a set of standardized tests to use, in order to do a preliminary evaluation on each patient, which would then hopefully direct the approach of treatment for each individual couple. This can be implemented when we have well designed studies that will help us to establish new protocols.
